# Supplementary material for: High-Fat Diets Modify the Proteolytic Activities of Dipeptidyl-Peptidase IV and the Regulatory Enzymes of the Renin–Angiotensin System in Cardiovascular Tissues of Adult Wistar Rats
Source: Biomedicines. 2021 Sep 3;9(9):1149. doi: 10.3390/biomedicines9091149 (PMC8470673; doi:10.3390/biomedicines9091149)
Supplement: Supplementary file 1 [file biomedicines-09-01149-s001.zip › Supplementary material-sup final.pdf]

Supplementary Table S1. Angiotensinase activities in atrium, ventricle, aorta and plasma.

Sample	Angiotensinase	Diet	Mean ± SEM (sol) [pmol/min/mg prot.]	P-value	Mean ± SEM (mb) [pmol/min/mg prot.]	P-value
Atrium	AspAP	S	Nd	-	59.99 ± 4.58	0.315
		VOO	Nd		53.13 ± 2.48	
		Bch	Nd		65.15 ± 8.16	
	GluAP	S	168.62 ± 17.71	0.901	789.70 ± 72.70	0.857
		VOO	153.51 ± 10.97		857.58 ± 84.48	
		Bch	166.22 ± 39.27		818.27 ± 105.56	
	AlaAP	S	584.10 ± 90.52	0.221	591.99 ± 72.12	0.744
		VOO	465.51 ± 75.83		521.35 ± 47.50	
		Bch	650.59 ± 75.67		531.41 ± 86.84	
	ArgAP	S	441.72 ± 80.25	0.576	582.15 ± 75.58	0.904
		VOO	404.10 ± 82.18		533.44 ± 64.48	
		Bch	499.56 ± 90.35		548.66 ± 96.41	
	CysAP	S	212.18 ± 32.74	0.042	201.40 ± 19.27	0.827
		VOO	195.50 ± 23.11#	#0.047	186.84 ± 13.70	
		Bch	310.75 ± 21.51*	*0.033	206.23 ± 31.40	
Ventricle	AspAP	S	10.14 ± 0.55	0.166	87.78 ± 3.76	0.934
		VOO	10.76 ± 1.23		86.61 ± 7.64	
		Bch	8.69 ± 0.40		84.84 ± 5.68	
	GluAP	S	49.29 ± 2.58	0.203	1034.23 ± 94.61	0.130
		VOO	49.85 ± 6.08		1026.31 ± 53.57	
		Bch	39.87 ± 3.30		1162.65 ± 35.85	
	AlaAP	S	221.62 ± 18.81	0.084	744.51 ± 71.48	0.486
		VOO	248.98 ± 50.01		739.20 ± 25.96	
		Bch	135.98 ± 27.46		812.91 ± 53.63	
	ArgAP	S	219.97 ± 25.71	0.039	856.11 ± 74.59	0.035
		VOO	255.20 ± 52.95	#0.025	763.06 ± 15.88#	#0.029
		Bch	127.80 ± 17.60#		1013.07 ± 56.57	
	CysAP	S	158.98 ± 8.20	0.491	401.45 ± 11.09	0.805
		VOO	195.70 ± 24.47		395.22 ± 16.67	
		Bch	160.06 ± 14.96		406.80 ± 5,80	
Aorta	AspAP	S	Nd	-	429.61 ± 30.98	0.253
		VOO	Nd		325.91 ± 13.34	
		Bch	Nd		417.64 ± 73.89	
	GluAP	S	7311.12 ± 1829.06	0.298	3538.40 ± 521.99	0.045
		VOO	6168.07 ± 1150.65		2024.36 ± 420.28#	#0.021
		Bch	9116.90 ± 1546.17		3986.00 ± 582.40	
	AlaAP	S	15,550.30 ± 2076.91	0.221	3542.19 ± 194.96	0.744
		VOO	13,923.76 ± 1763.02		3039.49 ± 307.43	
		Bch	19,133.57 ± 3485.98		3836.66 ± 315.52	
	ArgAP	S	12,872.48 ± 1807.96	0.337	1950.31 ± 125.24	0.037
		VOO	11,769.74 ± 1740.13		1548.75 ± 129.86#	#0.040
		Bch	15,948.30 ± 2238.67		2087.72 ± 139.98	
	CysAP	S	5576.68 ± 1040.81	0.453	1215.32 ± 105.63	0.578
		VOO	4701.84 ± 462.47		1069.51 ± 76.23	
		Bch	6266.42 ± 904.14		1148.11 ± 118.39	

Sample	Angiotensinase	Diet	Mean ± SEM [pmol/min/mL]	P-value
Plasma	AspAP	S	121.85 ± 12.22	0.649
		VOO	113.24 ± 11.97	
		Bch	127.44 ± 7.05	
	GluAP	S	153.91 ± 22.07	0.221

	VOO	113.24 ± 4.20	
	Bch	150.67 ± 13.55	
AlaAP	S	456.14 ± 85.43	0.756
	VOO	650.62 ± 228.71	
	Bch	506.14 ± 71.46	
ArgAP	S	332.63 ± 29.77	0.379
	VOO	284.66 ± 17.06	
	Bch	380.04 ± 30.76	
CysAP	S	288.80 ± 10.22	
	VOO	311.56 ± 38.63	0.932
	Bch	292.31 ± 10.39	

Note: The values represent means and standard error of the mean (SEM) of angiotensinases activities in plasma [pmol/min/mL] and soluble (sol) and membrane-bound (mb) fractions of atrium, ventricle and aorta [pmol/min/mg prot.]. AlaAP: alanyl aminopeptidase; ArgAP: arginyl aminopeptidase; AspAP: aspartyl aminopeptidase; CysAP: cystinyl aminopeptidase; GluAP: glutamyl aminopeptidase. Nd: Not detected. P-value less than 0.05 was considered significant. * $p < 0.05$, indicates significant differences between virgin olive oil diet (VOO) or butter plus cholesterol diet (Bch) vs. standard diet (S). # $p < 0.05$, indicates significant differences between VOO and Bch.

Supplementary Table S2. Dipeptidyl dipeptidase IV activity in atrium, ventricle, aorta and plasma.

Sample	Diet	Mean ± SEM (sol) [pmol/min/mg prot.]	P-value	Mean ± SEM (mb) [pmol/min/mg prot.]	P-value
Atrium	S	223.28 ± 35.25	0.808	1476.76 ± 102.34	0.035
	VOO	225.48 ± 41.31		1743.86 ± 99.46	#0.049
	Bch	254.23 ± 12.48		1222.50 ± 223.93#	
Ventricle	S	67.28 ± 5.65	0.095	3764.83 ± 163.21	0.200
	VOO	81.27 ± 14.14		4012.39 ± 205.71	
	Bch	48.92 ± 7.32		3550.59 ± 113.16	
Aorta	S	7490.30 ± 1347.70	0.150	3937.62 ± 478.42	0.033
	VOO	10,908.35 ± 991.10		3175.38 ± 102.01	*0.028
	Bch	10,596.26 ± 1542.62		2321.89 ± 224.54*	
**Sample**	**Diet**	**Mean ± SEM [pmol/min/mL]**	**P-value**		
Plasma	S	1605.29 ± 213.23	0.157		
	VOO	2536.39 ± 355.64			
	Bch	2051.97 ± 357.88			

Note: The values represent means and standard error of the mean (SEM) of dipeptidyl dipeptidase IV (DPP-IV) activity in plasma [pmol/min/mL] and soluble (sol) and membrane-bound (mb) fractions of atrium, ventricle and aorta [pmol/min/mg prot.]. P-value less than 0.05 was considered significant. * $p < 0.05$, indicates significant differences between virgin olive oil diet (VOO) or butter plus cholesterol diet (Bch) vs. standard diet (S). # $p < 0.05$, indicates significant differences between VOO and Bch.

Supplementary Table S3. Leucyl aminopeptidase, gamma-glutamyl transferase and pyroglutamyl aminopeptidase activities in atrium, ventricle, aorta and plasma.

Sample	Angiotensinase	Diet	Mean ± SEM (sol) [pmol/min/mg prot.]	P-value	Mean ± SEM (mb) [pmol/min/mg prot.]	P-value
Atrium	Leu	S	Nd	-	Nd	-
		VOO	Nd		Nd	
		Bch	Nd		Nd	
	GGT	S	Nd	-	Nd	-
		VOO	Nd		Nd	
		Bch	Nd		Nd	
	pGluAP	S	Nd	-	75.99 ± 1.82	0.008
		VOO	Nd		58.22 ± 4.92*	*0.011
		Bch	Nd		54.83 ± 6.00*	*0.030
Ventricle	Leu	S	291.37 ± 33.40	0.039	636.06 ± 70.51	0.004
		VOO	326.41 ± 69.34	*0.027	667.32 ± 23.08#	*0.005
		Bch	162.87 ± 21.53*		924.99 ± 39.31*	#0.013
	GGT	S	31.68 ± 2.58	0.233	244.02 ± 29.30	0.903
		VOO	32.36 ± 4.00		245.92 ± 22.14	
		Bch	23.57 ± 2.15		230.11 ± 24.83	
	pGluAP	S	10.27 ± 0.72	0.431	74.66 ± 1.52	0.173
		VOO	11.63 ± 1.37		80.41 ± 6.37	
		Bch	9.58 ± 1.11		77.16 ± 1.99	
Aorta	Leu	S	9667.46 ± 1561.22	0.198	2071.56 ± 100.69	0.006
		VOO	11,769.61 ± 1438.06		1753.36 ± 213.53#	#0.017
		Bch	14,058.63 ± 1713.46		2681.70 ± 111.30*	*0.025
	GGT	S	4543.64 ± 746.47	0.375	5473.36 ± 1047.74	0.972
		VOO	5739.25 ± 850.70		5266.88 ± 617.60	
		Bch	5031.80 ± 154.47		5555.47 ± 904.69	
	pGluAP	S	4717.07 ± 972.06	0.532	1145.60 ± 108.80	0.472
		VOO	3703.08 ± 411.05		977.09 ± 76.90	
		Bch	4487.61 ± 735.24		1000.01 ± 130.44	

Sample	Angiotensinase	Diet	Mean ± SEM [pmol/min/mL]	P-value
Plasma	Leu	S	236.58 ± 3.31	0.251
		VOO	246.10 ± 2.85	
		Bch	236.48 ± 3.75	
	GGT	S	122.02 ± 15.07	0.529
		VOO	101.47 ± 12.90	
		Bch	111.75 ± 8.85	
	pGluAP	S	108.22 ± 7.09	0.279
		VOO	99.37 ± 7.50	
		Bch	124.51 ± 15.37	

Note: The values represent means and standard error of the mean (SEM) of Leucyl aminopeptidase, gamma-glutamyl transferase and pyroglutamyl aminopeptidase activities in plasma [pmol/min/mL] and soluble (sol) and membrane-bound (mb) fractions of atrium, ventricle and aorta [pmol/min/mg prot.]. LeuAP: Leucyl aminopeptidase; GGT: gamma-glutamyl transferase; pGluAP: pyroglutamyl aminopeptidase activities. Nd: Not detected. P-value less than 0.05 was considered significant. * $p < 0.05$, indicates significant differences between virgin olive oil diet (VOO) or butter plus cholesterol diet (Bch) vs. standard diet (S). # $p < 0.05$, indicates significant differences between VOO and Bch.

Supplementary Figure S1. Group and intra-group correlations between significant glutamyl aminopeptidase, alanyl aminopeptidase, arginyl aminopeptidase, and cystinyl aminopeptidase activities analyzed in plasma and soluble and membrane-bound fractions of atrium, ventricle and aorta.

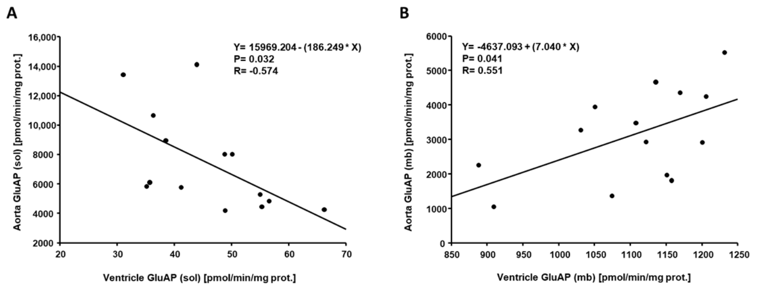

Intra-group correlation:	S – S	VOO – VOO	Bch - Bch
P-value:	0.441	0.171	0.519
R:	0,455	0,719	0,481

Intra-group correlation:	S – S	VOO – VOO	Bch - Bch
P-value:	0.046	0.556	0.471
R:	0,884	0,356	0,529

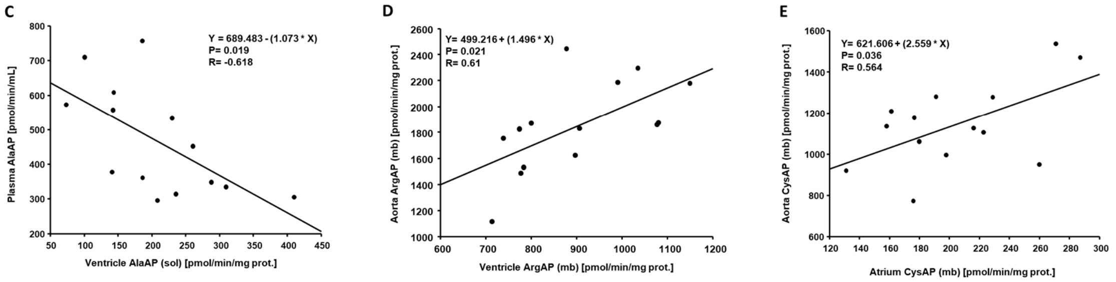

Intra-group correlation:	S – S	VOO – VOO	Bch - Bch
P-value:	0.461	0.167	0.537
R:	0,437	0,833	0,373

Intra-group correlation:	S – S	VOO – VOO	Bch - Bch
P-value:	0.146	0.231	0.334
R:	0,748	0,654	0,666

Intra-group correlation:	S – S	VOO – VOO	Bch - Bch
P-value:	0.011	0.957	0.569
R:	0,956	0,033	0,431

Supplementary Figure S1. Group and intra-group correlations between significant (A-B) glutamyl aminopeptidase (GluAP), (C) alanyl aminopeptidase (AlaAP), (D) arginyl aminopeptidase (ArgAP), (E) cystinyl aminopeptidase (CysAP) activities analyzed in plasma [pmol/min/mL] and soluble (sol) and membrane-bound (mb) fractions of atrium, ventricle and aorta [pmol/min/mg prot.]. P-value less than 0.05 was considered significant. R: linear correlation coefficient. The table below the figure represents the correlations between the same tissues with the same dietary groups (intra-group correlation).

Supplementary Figure S2. Group and intra-group correlations between significant dipeptidyl peptidase IV activity analyzed in plasma and soluble fractions of atrium.

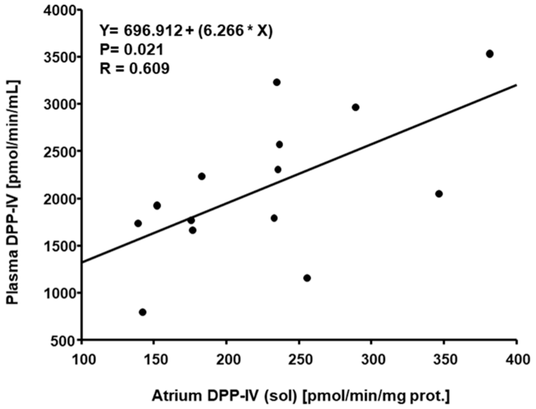

Intra-group correlation:	S – S	VOO – VOO	Bch - Bch
P-value:	0.734	0.051	0.717
R:	0,221	0,877	0,283

Supplementary Figure S2. Group and intra-group correlations between significant dipeptidyl peptidase IV (DPP-IV) activity analyzed in plasma [pmol/min/mL] and soluble (sol) fractions of atrium [pmol/min/mg prot.]. P-value less than 0.05 was considered significant. R: linear correlation coefficient. The table below the figure represents the correlations between the same tissues with the same dietary groups (intra-group correlation).

Supplementary Figure S3. Group and intra-group correlations between significant leucyl aminopeptidase activity analyzed in membrane-bound fractions of ventricle and aorta.

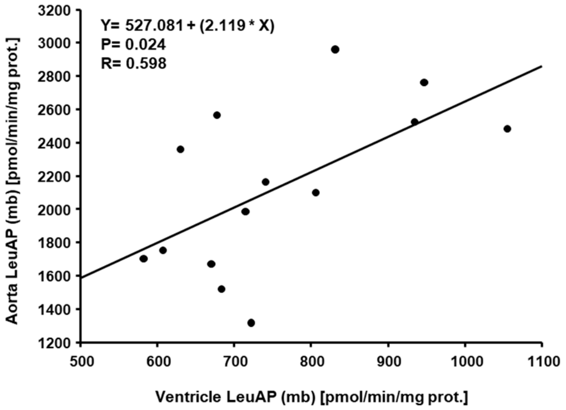

Intra-group correlation:	S – S	VOO – VOO	Bch - Bch
P-value:	0.288	0.806	0.151
R:	0.597	0.153	0.849

Supplementary Figure S3. Group and intra-group correlations between significant (leucyl aminopeptidase (LeuAP) activity analyzed in membrane-bound (mb) fractions of ventricle and aorta [pmol/min/mg prot.]. P-value less than 0.05 was considered significant. R: linear correlation coefficient. The table below the figure represents the correlations between the same tissues with the same dietary groups (intra-group correlation).
